# Characterizing the microbial metagenome of calcareous stromatolite formations in the San Felipe Creek in the Anza Borrego Desert

**DOI:** 10.1128/mra.00881-23

**Published:** 2024-03-04

**Authors:** Rosalina Stancheva, Arun Sethuraman, Hossein Khadivar, Jenna Archambeau, Ella Caughran, Ashley Chang, Brad Hunter, Christian Ihenyen, Marvin Onwukwe, Dariana Palacios, Chloe La Prairie, Nicole Read, Julianna Tsang, Brianna Vega, Cristina Velasquez, Xiaoyu Zhang, Elinne Becket, Betsy Read

**Affiliations:** 1 Department of Biological Sciences, California State University San Marcos, San Marcos, California, USA; 2 Department of Environmental Science and Policy, George Mason University, Fairfax, Virginia, USA; 3 Department of Biology, San Diego State University, San Diego, California, USA; 4 Department of Chemistry, American University, Washington, DC, USA; 5 Department of Biological Sciences, University of New Hampshire, Durham, New Hampshire, USA; 6 Department of Mathematics, Amherst College, Amherst, Massachusetts, USA; 7 Department of Biology, Howard University, Washington DC, USA; 8 Department of Biological Sciences, University of Maryland, Baltimore County, Maryland, USA; 9 Department of Biosciences, Farmingdale State College, Farmingdale, New York, USA; 10 Department of Biology, Millikin University, Decatur, Illinois, USA; 11 Department of Biological Sciences, Willamette University, Salem, Oregon, USA; Montana State University, Bozeman, Montana, USA

**Keywords:** stromatolite, metagenome

## Abstract

We describe the metagenome composition, community functional annotation, and prokaryote diversity in calcareous stromatolites from a dry stream bed of the San Felipe Creek in the Anza Borrego Desert. Analyses show a community capable of nitrogen fixation, assimilatory nitrate reduction, biofilm formation, quorum sensing, and potential thick-walled akinete formation for desiccation resistance.

## ANNOUNCEMENT

Stromatolites represent some of the earliest forms of marine cyanobacterial life, dating back 3.5 billion years (1, 2). Stromatolites are distributed from hypersaline coastal mats of the Hamelin Pools of Shark Bay in Western Australia (3) to oligotrophic lakes in the Chihuahuan Desert (4). Stromatolite freshwater habitats are extreme and exposed to desiccation events (5). We studied the temporally dry San Felipe Creek in the Anza Borrego Desert, Southern California, where stromatolites were previously identified (6).

**Fig 1:**
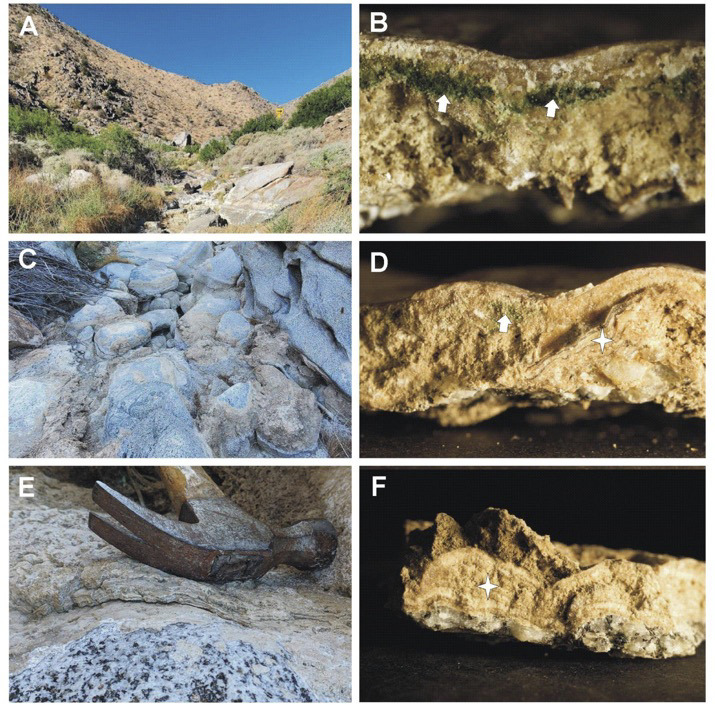
The ephemeral San Felipe Creek in Anza Borrego Desert (**A**). Samples were collected from the surface of boulders found in the bed of the desiccated creek (**C and E**). Samples imaged under a dissecting scope (**B, D, and F**) show characteristic features of stromatolites including endolithic cyanobacteria underneath a layer of calcite (arrows in panels B and D) and multiple laminate layers of organic and inorganic materials (asterisks in panels D and F). DNA was isolated from the stromatolites shown in panels B and E.

Materials were collected on 20 November 2019 from granite rock tops in the San Felipe Creek (Fig. 1) (33.0986,–116.4708) (7). A single, dry stromatolite (2 cm thick) was chiseled, transported to California State University, San Marcos (CSUSM), and material (500 mg) from the entire sample, without targeting any specific layer, was ground with a bead mill. Cells were extracted with PEG-NaCl buffer prior to isolating DNA (8). Four replicate metagenomic libraries were prepared from a single stromatolite using the TELL-Seq WGS Library Prep Kit (Universal Sequencing, Carlsbad, CA, USA). Pooled libraries were sequenced on Illumina NextSeq 500/550 platform using the Mid Output Kit V2.5 (150 cycles, paired-end reads). Raw reads (FASTQ) were assessed using FastQC v.11.9 (*Q* score ≥ 30), and *de novo* metagenomes were assembled with UST TELL-Seq assembly pipeline. Metagenome quality was assessed with QUAST v.4.4 (8) on KBase v.2.6.4 (9). Kaiju v.1.7.3 (10) against the NCBI microbial genomes database in KBase v.2.6.4 (9) was used to classify operational taxonomic units (OTUs) from normalized raw reads (11). Automated gene calling was performed using NCBI PGAP (12) with *Lyngbya aestuarii* and *Sediminibacterium* as references. Predicted proteins were classified using GhostKOALA v.2.2 (13).

Pooled sequencing generated ~2.05 × 10^7^ reads with >90.6% exceeding *Q* ≥ 30 and having a GC content of ~54% GC. While 2.8 Gb of sequence data was generated from each replicate library, the average metagenome assembly was 1.3 Mb (10× depth). Optimized assembly with pooled reads yielded a larger, contiguous metagenome (~29 Mb). The largest scaffold was ~1.1 Mb with an *L*_50_ count of 109 and *N*_50_ size of 56 Kb (Table 1).

**TABLE 1:** Quality metrics were performed on the combined metagenomics data using QUAST and show a relatively large and contiguous 29 Mb metagenome

Parameter/statistic genome assembly	Metagenome
Number of scaffolds	5,478
Total scaffold size (Mb)	29.03
Longest scaffold (Mb)	1.13
Shortest scaffold (bp)	500
Number of scaffolds >1,000 nt	2,788 (50.9%)
Number of scaffolds >10,000 nt	355 (6.5%)
Number of scaffolds >100,000 nt	38 (0.7%)
Number of scaffolds >1,000,000 nt	1 (0.0%)
Number of scaffolds >10,000,000 nt	0 (0.0%)
Mean scaffold size (bp)	5,300
Median scaffold size (bp)	1,021
*N*_50_ scaffold length (bp)	56,004
*L*_50_ scaffold count	109
%GC	41.44

Genome annotation	
Genes (total)	33,494
Coding Sequences (CDS with protein homology)	33,203
Genes (RNA)	291
rRNAs	7, 16, 34 (5S, 16S, 23S)
tRNAs	221
ncRNAs	13
Pseudogenes (total)	1,127
CRISPR	65

Classification of raw read OTUs showed that the prokaryotic communities were primarily composed of Cyanobacteria (85.95%, 45 genera), followed by Proteobacteria (5.73%) and Actinobacteria (4.33%). The taxonomic composition of Anza Borrego stromatolites was similar to freshwater stromatolites from pools in Cuatros Cienegas Basin (14) and Ruidera, Spain (5), which were also dominated by cyanobacteria (74% and 54%, respectively).

Gene prediction using PGAP resulted in community-level annotation of ~33,000 protein-coding genes (Table 1), including 7 complete 5S rRNA, 16 16S rRNA, and 34 23S partial rRNA genes. Of these, 11,732 (37%) were classified, 10% being affiliated with signaling and cellular processes like nitrogen cycling, quorum sensing, biofilm formation, and desiccation resistance. Additionally, several genes were linked to genetic information processing (9%), carbohydrate metabolism (9%), and environmental information processing (9%). Metagenomic signatures for morphological and physiological strategies to cope with the harsh conditions of the Anza Borrego Desert were found, including those for UV protective pigments scytonemin and carotenoids, and the synthesis of potential mycosporine-like amino acids; genes involved in microalgal desiccation tolerance, including those encoding aquaporins, chaperones, and antioxidants; and enzymes responsible for trehalose, sucrose, and polyamine synthesis.

## Data Availability

The assembled metagenome has been submitted to NCBI and is accessible via BioProject: PRJNA967693. This Whole Genome Shotgun project has been deposited at DDBJ/ENA/GenBank under the accession JASEJY000000000. The version described in this paper is version JASEJY010000000. Scripts and code for all analyses performed can be accessed via the project’s GitHub page, accessible at https://github.com/j-archambeau/Stromatolite_Metagenomics
